# Connexin 43 Is a Potential Prognostic Biomarker for Ewing Sarcoma/Primitive Neuroectodermal Tumor

**DOI:** 10.1155/2011/971050

**Published:** 2011-05-08

**Authors:** Marilyn M. Bui, Gang Han, Geza Acs, Damon Reed, Ricardo J. Gonzalez, T. L. Pasha, Paul J. Zhang

**Affiliations:** ^1^Department of Anatomic Pathology, Moffitt Cancer Center, Tampa, FL 33612, USA; ^2^Program of Experimental Therapeutics, Moffitt Cancer Center, Tampa, FL 33612, USA; ^3^Biostatistics Core, Moffitt Cancer Center, Tampa, FL 33612, USA; ^4^Department of Sarcoma, Moffitt Cancer Center, Tampa, FL 33612, USA; ^5^Department of Pathology & Laboratory Medicine, Hospital of the University of Pennsylvania, Philadelphia, PA 800-789, USA

## Abstract

Connexins (Cxs) are building unit proteins of gap junctions (GJs) that are prognostic markers in carcinomas. To investigate the role of Cx in Ewing sarcoma (EWS)/primitive neuroectodermal tumor (PNET), we examined the expression of Cx43 and Cx26 in 36 EWS/PNETs and found (1) cytoplasmic Cx43 reactivity in 28/36 (78%) cases. (2) Cx43 score was significantly correlated with overall survival (*P* = .025). The average scores for patients alive and dead at 3 years are 46.08 and 96.98 (*P* = .004) at 5 years are 46.06 and 96.42 (*P* = .002). (3) Metastasis had a significant effect on the overall survival (*P* = .003). (4) Cytoplasmic Cx26 reactivity was detected in 2 of 36 (6%) patients who died with metastasis. Our results suggest a possible oncogenic and prognostic role for Cx43 and Cx26 in EWS/PNET. The lack of membranous immunoreactivity suggests that the effect of Cx in EWS/PNET is via a GJ function-independent mechanism.

## 1. Introduction

Ewing sarcoma (EWS)/primitive neuroectodermal tumor (PNET) is an aggressive sarcoma that commonly affects children and young adults. It is primarily a tumor of bone but may also develop in soft tissue [1]. The pathogenesis and histogenesis of EWS/PNET is largely unknown, but the vast majority of patients have the 11 : 22 EWS-FLI-1 translocation. The prognosis of patients who develop advanced disease remains poor. The current available prognostic biomarkers for this group of tumors are very limited. Discovery of novel biomarkers that have prognostic and predictive value for patients with EWS/PNET would serve as guidance for the development of novel targeted therapeutic strategies. 

Connexins (Cxs) are a family of homologous proteins that serve as the building blocks of gap junctions (GJs). GJs permit the direct exchange of small molecules between cells. GJs are present in all types of vertebrate cells, except red blood cells, platelets, some neurons, and spermatozoids [2] and represent a fundamental structure necessary for normal cell function [3]. The Cx-mediated GJ communication also plays a critical role in osteoblast differentiation [3–5]. Among the 20 known Cxs, Cx43, Cx45, and Cx46 are expressed in bone. Cx43 is the primarily expressed form and plays major and diverse roles in bone development [5]. In addition to the important role of Cx43 in bone development and differentiation, dysregulation of Cx expression is believed to have a role in carcinogenesis. Cxs, especially Cx43 and Cx26, have been shown to be associated with carcinomas of the lung, breast, prostate, liver, stomach, and colon [6–14]. Cxs were also shown to be involved in invasion and metastasis of melanoma and acute leukemia [9, 13]. However, little is known with regard to the role of Cx in sarcomas. In this study, we examined the expression of Cx43 and Cx26 in a series of EWS/PNET and correlated the results with various clinicopathologic features and patient outcome in order to explore their potential role in the biology of this group of sarcomas. 

## 2. Materials and Methods

This study was carried out in accordance with a research protocol approved by the Scientific Review Committee of the Moffitt Cancer Center and the Institutional Review Board of the University of South Florida.

### 2.1. Tissue Samples and Tissue Microarray (TMA)

A retrospective review was conducted to identify cases of EWS/PNET diagnosed between 1995 and 2007 and archived at the Department of Anatomic Pathology at the Moffitt Cancer Center. All cases were reviewed and diagnosis confirmed by a sarcoma pathologist (MMB). Representative formalin-fixed, paraffin-embedded tumor blocks were selected for tissue microarray (TMA) construction at the Histology Core Facility of the Moffitt Cancer Center [15]. The corresponding H&E slides of the TMA were reviewed to determine tissue integrity prior to immunohistochemical testing. 

### 2.2. Patient Data

Pertinent clinical data of patients were compiled from two sources: (1) pathology database to include age, sex, tumor location, tumor size, and ancillary study results and (2) tumor registry to include treatment and survival information. All patients were treated at the Moffitt Cancer Center with neoadjuvant chemotherapy, followed by resection. External beam radiation therapy was reserved for resections with positive surgical margins or unresectable disease.

### 2.3. Immunohistochemistry (IHC) and Image Analysis

Four-*μ*m-thick sections from the TMA were used for immunohistochemical staining for Cx43 (goat polyclonal, CXN-6, 1 : 250 dilution, Santa Cruz Biotechnology Inc., Santa Cruz, CA) and Cx26 (rabbit polyclonal, 1 : 100 dilution, Zymed Laboratories, San Francisco, CA). Antigen retrieval was performed in 10 mmol/L of sodium citrate buffer (pH 7.6) in a microwave oven for 4 minutes twice at 70% power level. Endogenous peroxidase was blocked by incubation in 5% hydrogen peroxide for 5 minutes. Nonspecific binding sites were blocked by incubating with 2% normal horse serum for 20 minutes. Sections were incubated with the primary antibodies at room temperature for 60 minutes. Immunoreactivity was visualized by using the DAKO EnVision+ System, HRP labeled polymer on a DAKO autostainer (DAKO, Carpinteria, CA). Both a positive and a negative control were used according to the manufacture's recommendation. The controls were brain tissue. The negative control was run without the primary antibody.

Immunoreactivity was quantitatively evaluated by automated slide scanning using the Aperio ScanScope XT (Vista, CA) and Image Pro Plus v6.2 (Bethesda, MD) analyzing macroalgorithms based on the intensity (0–3) and percentage (%) of staining. A score of 0–300 was calculated for each case as the product of the intensity score and the percent of immunoreactivity. The image analysis result was then quality controlled by a sarcoma pathologist (MMB) to determine the cutoff for positive verses negative stain.

### 2.4. Statistical Analysis

Median Cx immunostaining scores were compared between patients with metastatic disease and localized disease, as well as between patients alive and dead at 3 years and 5 years using the Mann-Whitney-Wilcoxon (MWW) test. Overall survival distributions of patients with metastatic disease and localized disease were visualized using the method of Kaplan-Meier curve. The log-rank test was used to test the effect of metastasis on overall survival. The proportional hazard model was used to test the effects of median Cx immunostaining scores and metastasis on the overall survival. The point estimation of the hazard ratio and the *P* value (from the test of whether the hazard ratio is equal to 1) was reported. In our analysis, a *P*-value less than  .05 was considered as being statistically significant. Computations were performed using the Statistical Analysis System (SAS), Version 9.2 (SAS Institute, Cary, NC) software.

## 3. Results

### 3.1. Cx Expression Pattern

**Figure 1:**
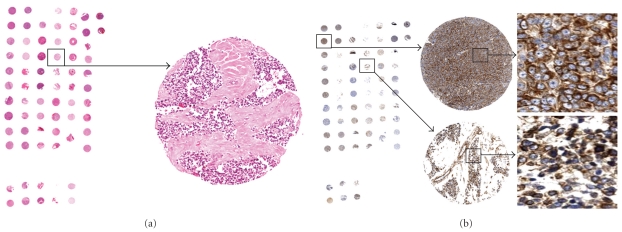
EWS/PNET TMA H&E and Cx43 stains. (a) Digital scan image of H&E Ewing TMA. Each patient has duplicate representative sections of the tumor. The enlargement is an example of a tumor section which is composed of small blue round cells with fibrous/hyalinized stoma. (b) Digital scan image of Cx43 immunostain. Two examples of the tumor sections showing cytoplasmic immunoreactivity.

The Cx43 expression is illustrated in Figure 1. The score of Cx43 is listed in Table 1. A score of less than 15 was considered as negative stain. Although there is no published benchmark for the cutoff for connexin, a score of less than 15 and microscopically immunoreactive in less than 5% tumor cells with only 1-2+ intensity stain was defined as negative. A score of 15 and microscopically 5% tumor cells with 3+ intensity stain was considered positive. We did not encounter a case that was less than 5% tumor cells with 3+ stain intensity. The pathologists involved in this study agreed to this cutoff. By image analysis, the range of the immunoreactivity was from 1.24 to 160.38. Cases considered negative ranged from no to very weak and focal immunoreactivity by microscopic examination. Whole section stains of 4 cases were done. Two were positive and 2 were negative which were consistent with the corresponding TMA findings. One of the two positive cases revealed heterogeneous staining pattern. Due to the heterogeneity of the tumor, we did not expect the two cores for each case to always be the same; therefore, an average score was used for final correlation calculation. Cytoplasmic Cx43 reactivity was detected in 28/36 (78%) cases (median score 62, range 1–160). Cytoplasmic Cx26 reactivity was observed in 2 of 36 (6%) cases.

### 3.2. Clinicopathological Correlation

The pertinent clinicopathological data are summarized in Table 1. Among the 36 patients, 19 were alive and 17 were dead of disease at their followup days (median followup 410 days). Twelve (33.3%) patients had metastasis at presentation and were analyzed separately from the remaining localized patients due to the different prognoses between these groups.

### 3.3. Statistical Analysis

The statistical analysis was done for the entire group of patients. The median survival time for all the patients is 1929, days (which is about 5 years) with 95% confidence interval from 701 days to infinity. 

**Figure 2:**
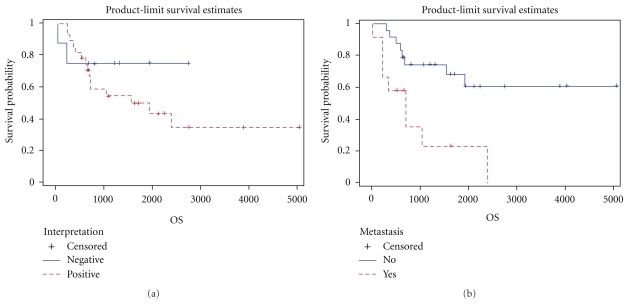
The Kaplan-Meier estimates of overall survival by (a) Cx43 interpretation; (b) metastasis.

We implemented the Cox proportional hazard model and estimated the hazard ratio of the Cx43 score to test the effect of the score of Cx43. The estimated hazard ratio of score is 1.019, and the *P*-value is  .004 indicating that higher score of Cx43 corresponds to significantly larger failure rate. Besides using Cx43 score, we tested the effect of the interpretation of Cx43.  Figure 2(a) depicts the Kaplan-Meier estimate of the survival function of the positive and negative interpretation groups. The log-rank test gives a nonsignificant *P*-value  .360, which indicates that the dichotomized interpretation is not significant, and that given the significance of the Cx43 score, this dichotomization can lower the test power. Due to the high censoring rates (46.4% for the positive and 75% for the negative) and the limited numbers of observation (28 positive and 8 negative patients), finite interval estimates of the two corresponding median survival times were not available. 

We compared the Cx43 scores for patients alive and dead at 3 years, as well as at five years using the MWW test. The two average scores of patients who were alive and dead at 3 years were 46.08 and 96.98, respectively, which were significantly different with *P*-value  .004. A similar result was found for 5-year survival. The average score of patients who were alive and dead at 5 years were 46.06 and 96.42, respectively, which were significantly different with *P*-value  .002. 

We studied the effect of metastasis on the overall survival, and the association between metastasis and Cx43 score.  Figure 2(b) shows the Kaplan-Meier curves for the patients with metastatic disease and localized disease. The curve for patients with metastasis is lower than the other at all the time points. Comparing the overall survival distributions of patients with and without metastasis, log-rank test gave *P*-value  .003 which indicates statistical significance in metastasis. The hazard ratio between patients without and with metastasis was 0.25, with *P*-value  .006. This result indicates that the patient group with metastasis had significantly higher failure rate compared to the group without metastasis. We further compared the distributions of Cx43 score for patients with and without metastasis. The *P*-value derived from the MWW test is  .606 indicating that *no* significant difference was found in Cx43 score between patients with and without metastasis. 

We conducted multivariable analysis by including both metastasis and Cx43 score. The hazard ratio for metastasis is 0.29 with *P*-value  .015. The hazard ratio for Cx43 score is 1.018 with *P*-value  .009. The effects of both metastasis and Cx43 score in this multivariable analysis are comparable to their effects in the aforementioned univariable analyses. This indicated that the effect of metastasis on overall survival is significant in addition to the effect of Cx43 score and vice versa.

 For the 2 cases showing Cx26 positivity, the patients died with metastasis to lung and brain at 341 days and 211 days after diagnosis, respectively.

## 4. Discussion

GJs function by transferring information between neighboring cells in the form of a secondary messenger (such as calcium) following a primary stimulus [3]. Cx43 is ubiquitous in all cells, and it is the predominant GJ protein in bone cells [16]. Cx43 serves both gap junction-dependent and -independent functions and plays a significant role in controlling bone formation, differentiation, and development [3–5, 16]. Furthermore, previous studies suggested that aberrant cytoplasmic localization and disturbance of GJ intercellular communication play an important role in carcinogenesis, invasion, and metastasis in some human malignancies including carcinomas, melanoma, and leukemia [6–14]. The role of Cxs (Cx43 in particular) in sarcoma remains unknown. Although a few studies of Cx43 in human sarcoma cell lines have been reported, which include osteosarcoma, rhabdomyosarcoma, and fibrosarcoma [17–21], there has not been any studies reported on the expression of Cx43 in formalin-fixed and paraffin-embedded tissue samples of human EWS/PNET.

Our study shows that EWS/PNET expresses cytoplasmic Cx43 frequently (78%). A higher level of Cx43 expression was correlated with adverse outcome and shorter survival in EWS/PNET patients, regardless of their stage, location, tumor size, and clinical management. In contrast to Cx43, Cx26 was rarely detected in EWS/PNET with only 2 of 36 (6%) cases showing cytoplasmic Cx26 immunoreactivity. In both cases, patients died with metastasis in 341 days and 211 days after diagnosis, respectively. 

The finding of cytoplasmic Cx expression in EWS/PNET is of interest. Cx is normally expressed on cell surface as membranous proteins that build blocks of GJs which function in regulating cell proliferation and apoptosis [8]. Normal expression of Cx plays important role in maintaining normal GJ function and regulating cell proliferation [8]. Studies have shown that in carcinomas, lack of functional intercellular connections is reflected by aberrant cytoplasmic accumulation of Cxs. Because of the proteins of gap junction channels, components are stored in the cytoplasm, and dysfunctional trafficking decreases the uptake of Cxs by the cell membrane from the cytoplasm [8, 22, 23]. Since the normal tissue counterpart of Ewing sarcoma is unknown, this study was not able to investigate if there is a “normal” expression pattern of Cx43 in Ewing cells. However, studies of human fibrosarcoma and osteosarcoma cell lines demonstrate that the expression/distribution pattern of Cx43 varies with different experimental conditions suggesting that aberrant pattern be cytoplasmic or nuclear [17–20]. The abnormal or aberrant expression might be related to a defect in GJ assembly associated with increased Cx synthesis and/or Cx degradation. In addition, this abnormal or aberrant expression seen with immunohistochemical technique is not uncommon in other oncoproteins. For example, C-kit is expected to be membranous staining in normal circumstance; however, cytoplasmic and globular/dot-like stain patterns are frequently encountered on routine immunohistochemical stain and biologically the same as membranous staining [24]. 

In addition to its GJ-dependent function, Cx has also been shown to have biologic functions independent of GJs in colorectal cancer [25–28]. As Cx43 is a downstream target of *β*-catenin, a key component of Wnt signaling pathway, nuclear accumulation of *β*-catenin turns on several genes including Cx43, COX-2, cyclin-D1, and PPAR*δ* and contributes to the carcinogenesis of colon cancer [25–28]. Cx has been shown to have a tumor suppressor role in experimental setting in some carcinomas [7, 8], whereas it seems to function as an oncoprotein in others [28]. Saito et al. [21] have shown that Cx43 can suppress the proliferation of U2OS osteosarcoma cells by increasing the level of p27 protein via posttranscriptional regulation. Krutovskikh et al. [22] have demonstrated that negative growth control of osteosarcoma cell by Bowman-Birk protease inhibitor from soybean involves Cx43. Therefore, the role of Cxs in tumor biology appears to be more complex than what was once believed and likely multifaceted. The lack of membrane reactivity and presence of cytoplasmic reactivity suggest that these Cxs likely function in a GJ-independent mechanism in EWS/PNET. 

 Our finding of frequent Cx43 cytoplasmic expression in EWS/PNETs and higher expression in tumors showing a more aggressive clinical behavior also suggests a potential oncogenic role of Cx43 in these tumors. As most of the EWS/PNETs in this study were confirmed to have EWS-FLI1 translation, Cx43 or Cx26 expression is not affected by EWS-FLI1. Although the data is limited, our finding of cytoplasmic Cx26 expression in only two cases with distant metastasis and poor outcome suggests that Cx26 cytoplasmic expression may be a rarer event, potentially a more advanced secondary event in a subset of EWS/PNET. 

Our study has shown that the score of Cx43 was significantly correlated with overall survival; however, there was no association of Cx43 score with metastasis, probably due to the limited sample size of 36. For our dataset, both Cx43 score and metastasis have been proven to be important prognostic factors for overall survival. 

Our findings suggest that immunohistochemical detection of Cx might provide prognostic information in this group of patients for whom there is very limited prognostic biomarkers currently available. Currently, EWS is treated uniformly with a combination chemotherapeutic regimen without dose escalation or reduction based on presenting features for localized disease. Intensification of therapy with autologous hematopoetic stem cells is being explored in limited metastatic settings. Though the level of Cx43 cytoplasmic expression would not be proposed to change therapy at this time, it can be further studied in larger series as a potential prognostic marker. Understanding the role of aberrant Cx43 expression in EWS/PNET may help explain the ontogeny of EWS, identify an important step in oncogenesis in a subset of tumors, and eventually serve as a therapeutic target.

Given the important role of Cx43 in tissue development, differentiation, and carcinogenesis, especially in bone cells, alterations in the expression of Cx43 may influence cell-cell communication and may serve as a potential prognostic marker as well as a target for novel agents in Ewing sarcoma. Given that the sample size of this study is limited (36 patients in total), power of the statistical tests can be improved with more patients, and the interaction effects of the independent variables can be investigated in the survival analysis as well. More studies and larger sample sizes are needed to further investigate the potential prognostic/predictive role of Cx in EWS/PNET.

## Figures and Tables

**Table 1:** The pertinent pathological, clinical, and Cx43 immunohistochemical data.

Case	Sex	Age	Location	Tumor size (cm)	Cx43 score	Cx43 interpretation	Metastasis	Treatment	OS	VS
1	M	24	Chest wall	10.5	160.38	Positive	No	S, C, R	610	Dead
2	M	17	Lower lobe lung	21.5	112.45	Positive	No	C, R	623	Dead
3	F	72	Thigh	6	117.59	Positive	No	S, C, R	298	Dead
4	M	28	Pelvis	14	8.62	Negative	Yes	C, R	222	Dead
5	M	30	Thigh	17	95.83	Positive	No	S, C, R	2743	Alive
6	F	28	Femur	12	63.19	Positive	Yes	S,C, R	522	Alive
7	M	40	Pelvis	10	18.49	Positive	No	S, C, R	1700	Alive
8	M	17	Pelvis	N/A	55.05	Positive	Yes	C, R	1029	Dead
9	M	24	Pelvis	12.7	60.17	Positive	No	S, C, R	1067	Alive
10	M	59	Thigh	4.5	110.12	Positive	Yes	S, C	1624	Alive
11	F	15	Shoulder/Humerus	5.3	55.09	Positive	No	S, C	4036	Alive
12	M	33	Spine (T7)	1.6	140.11	Positive	Yes	S, C, R	209	Dead
13	F	15	Femur	3.7	126.51	Positive	Yes	S, C	701	Dead
14	F	57	Leg	34.5	144.65	Positive	No	S, C	505	Dead
15	M	12	Distal femur	11	72.41	Positive	No	S, C, R	5065	Alive
16	M	35	Flank	3	83.9	Positive	No	S, C, R	3892	Alive
17	M	58	Thigh	8	56.45	Positive	Yes	S, C, R	661	Alive
18	F	35	Fibula	7	5.3	Negative	No	S, C, R	1301	Alive
19	M	54	Chest	8	135.54	Positive	No	S, C, R	681	Dead
20	M	15	Ilium	7.5	78.71	Positive	No	S, C, R	1929	Dead
21	M	28	Rib	5	88.57	Positive	No	S, C, R	1553	Dead
22	F	61	Chest	7.5	73.89	Positive	No	S, C	364	Dead
23	M	16	Pelvis	7.5	12.71	Negative	No	S, C, R	1929	Alive
24	F	16	Rib	5	50.25	Positive	No	S, C	2233	Alive
25	M	14	Leg	N/A	16.61	Positive	No	S, C	637	Alive
26	F	71	Uterus	11	13.07	Negative	Yes	S	25	Dead
27	M	29	Thigh	30	14.43	Negative	No	S, C, R	2743	Alive
28	F	41	Lung	8	17.25	Positive	Yes	S, C, R	2388	Dead
29	M	34	Chest wall	5.7	32.34	Positive	No	S, C	1624	Alive
30	F	67	Lung	7	109.74	Positive	Yes	S, C, R	341	Dead
31	F	20	Brain	1.2	1.24	Negative	No	S, C, R	806	Alive
32	F	16	Rib and diaphragm	5	104.5	Positive	Yes	S, C, R	711	Dead
33	M	58	Abdominal wall	5.5	2.42	Negative	No	S, C, R	1204	Live
34	M	37	Brain	2	2.94	Negative	No	S, R	664	Live
35	M	24	Cerebellum	1	75.33	Positive	No	S, R	2109	Live
36	F	47	Brain	2	55.68	Positive	Yes	S, C, R	211	Dead

*S: Surgery; C: Chemotherapy; R: Radiation, OS: Overall survival rate; VS: Vital status.
